# Cover of coastal vegetation as an indicator of eutrophication along environmental gradients

**DOI:** 10.1007/s00227-016-3032-6

**Published:** 2016-11-21

**Authors:** Sofia A. Wikström, Jacob Carstensen, Mats Blomqvist, Dorte Krause-Jensen

**Affiliations:** 1 Baltic Sea Centre, Stockholm University, 10691 Stockholm, Sweden; 2 Department of Bioscience, Aarhus University, Frederiksborgvej 399, 4000 Roskilde, Denmark; 3 Hafok AB, 179 61 Stenhamra, Sweden; 4 Department of Bioscience, Aarhus University, Vejlsøvej 25, 8600 Silkeborg, Denmark

## Abstract

Coastal vegetation communities are important for primary production, biodiversity, coastal protection, carbon and nutrient cycling which, in combination with their sensitivity to eutrophication, render them potential indicators of environmental status for environmental policies like the EU Water and Marine Strategy Framework Directives. We evaluated one potential indicator for coastal vegetation, the cumulative cover at depths where the vegetation is light limited, by investigating its response to eutrophication along gradients in natural conditions. We used a large data set covering the Swedish coastline, spanning broad gradients in nutrient level, water clarity, seabed substrate, physical exposure and climate in addition to a salinity gradient from 0.5 to 30.5. Macroalgal cover increased significantly along gradients of declining nutrient concentration and increasing water clarity when we had accounted for diver effects, spatio-temporal sampling variability, salinity gradients, wave exposure and latitude. The developed empirical model explained 79% of the variation in algal cover across 130 areas. Based on this, we identified macroalgal cover as a promising indicator across the Baltic Sea, Kattegat and Skagerrak. A parallel analysis of soft-substrate macrophytes similarly identified significant increases in cover with decreasing concentrations of total nitrogen and increasing salinity, but the resulting empirical model explained only 52% of the variation in cover, probably due to the spatially more variable nature of soft-substrate vegetation. The identified general responses of vegetation cover to gradients of eutrophication across wide ranges in environmental settings may be useful for monitoring and management of marine vegetation in areas with strong environmental gradients.

**Electronic supplementary material:**

The online version of this article (doi:10.1007/s00227-016-3032-6) contains supplementary material, which is available to authorized users.

## Introduction

Macroalgae and seagrasses form vegetated belts along the world’s coastlines (Gattuso et al. 2006). These belts increase the structural complexity and change the physico-chemical environment, facilitating colonization of other species and thereby promoting biodiversity in the coastal zone (Gutiérrez et al. 2011). They provide shelter for a variety of species and are important primary producers; their metabolism markedly affect the cycling of carbon and nutrients, and through enhanced sedimentation and stabilizing water flow, they contribute to protecting sandy coasts from erosion while also promoting water clarity (Jones et al. 1994; Hemminga and Duarte 2000; Orth et al. 2006; Gutiérrez et al. 2011). These key functions and ecological services make seagrass meadows and macroalgal beds rank among the most valuable ecosystems of the world (Costanza et al. 1997; Barbier et al. 2011). Ensuring extended vegetation cover and assessing its status is, therefore, of key importance in coastal management and monitoring.

The rapid growth of the human population and the concentration of people and activities along the shores (Nicholls and Small 2002) have caused reductions in coastal water quality (Nixon and Fulweiler 2009) and pose threats to coastal ecosystems including the coastal vegetation (Lotze et al. 2006; Waycott et al. 2009). These challenges have prompted environmental policies such as the European Water Framework Directive (WFD, 2000/60/EC) and the European Marine Strategy Framework Directive (MSFD, 2008/56/EC) directed at assessing the status and ensuring a good quality of coastal ecosystems through management action. Consequently, it is important to identify and document good indicators of coastal quality, including benthic vegetation indicators. Central criteria for good indicators are ecosystem relevance and scientific basis for response to pressures, and large-scale applicability is also an asset (ICES 2013; Queirós et al. 2016).

The response of coastal vegetation to impaired water quality includes changes in vegetation cover or abundance and shifts in species composition (Duarte 1995). Accordingly, the WFD defines good ecological status for coastal vegetation as “most disturbance sensitive macroalgal and angiosperm taxa associated with undisturbed conditions are present and the level of macroalgal cover and angiosperm abundance show slight signs of disturbance” (WFD, 2000/60/EC). This means that it is important to monitor vegetation cover as an indicator for the status of coastal vegetation and identify factors regulating the cover.

Increased nutrient concentrations can result in excessive growth of opportunistic macroalgae, leading to high algal abundance in shallow waters (e.g. Valiela et al. 1997). Furthermore, nutrients stimulate phytoplankton growth resulting in more turbid waters and less light reaching the sea bed. Below a certain depth, light is the key regulating factor for growth of benthic vegetation, which means that increased nutrient concentrations and reduced water clarity lead to reductions in vegetation cover (e.g. Pedersen and Snoeijs 2001; Krause-Jensen et al. 2003; Krause-Jensen et al. 2007a). The fact that the cover of the entire vegetation community can respond differently to eutrophication at different depths makes it important to take water depth into account when attempting to use cover as indicator for anthropogenic nutrient enrichment. In addition, a number of other natural gradients can affect vegetation cover. For instance, physical exposure to wind and waves can stimulate vegetation growth by increasing turbulence and nutrient transport or cause loss of biomass and reductions in cover, particularly in shallow waters where physical perturbations are strong (Hurd 2000; Koch 2001). Despite this, few studies have evaluated how the relationship between vegetation cover and eutrophication-related variables is affected by large-scale natural gradients in environmental variables.

The Swedish coastline represents major gradients in environmental conditions; the salinity declines from close to oceanic levels in Skagerrak to almost freshwater in the Bothnian Bay, and there are large differences in, for example, eutrophication, seabed substrate, water temperatures and levels of physical exposure, both within and between regions. These small- and large-scale differences create strong gradients in vegetation composition. For instance, the decline in salinity is paralleled by a steep decrease in the number of macroalgal species (Nielsen et al. 1995; Middelboe et al. 1997). Soft-substrate vegetation shows the opposite pattern with generally monospecific eelgrass (*Zostera marina*) meadows in the most saline areas and an increasing contribution of other vascular plants and charophytes in mixed-species meadows in more brackish regions (Selig et al. 2007; Boström et al. 2014). Such changes in vegetation composition along natural gradients can be expected to affect also vegetation cover and possibly the response of the vegetation to eutrophication. Previous studies have shown reductions in cover of macroalgae and soft-substrate vegetation due to increased nutrient concentrations and reduced water clarity in local areas of the Baltic Sea and Kattegat (Krause-Jensen et al. 2003; 2007a, b, 2009; Hansen and Snickars 2014). However, no studies have explored how the cover of macroalgae or soft-substrate vegetation respond to eutrophication pressure along broad gradients of salinity, exposure and climatic conditions such as those present across the Baltic Sea, Kattegat and Skagerrak.

This study aims to test the hypothesis that the response of coastal vegetation to eutrophication pressure is uniform over natural gradients in salinity, exposure and climate variables, when these gradients are accounted for. We test the hypothesis on a large monitoring data set of vegetation cover along the entire Swedish coastline spanning latitudes from 55.4 to 65.8°N and representing wide gradients in eutrophication as well as in salinity, physical exposure, light and temperature.

## Methods

### Study areas

**Fig. 1:**
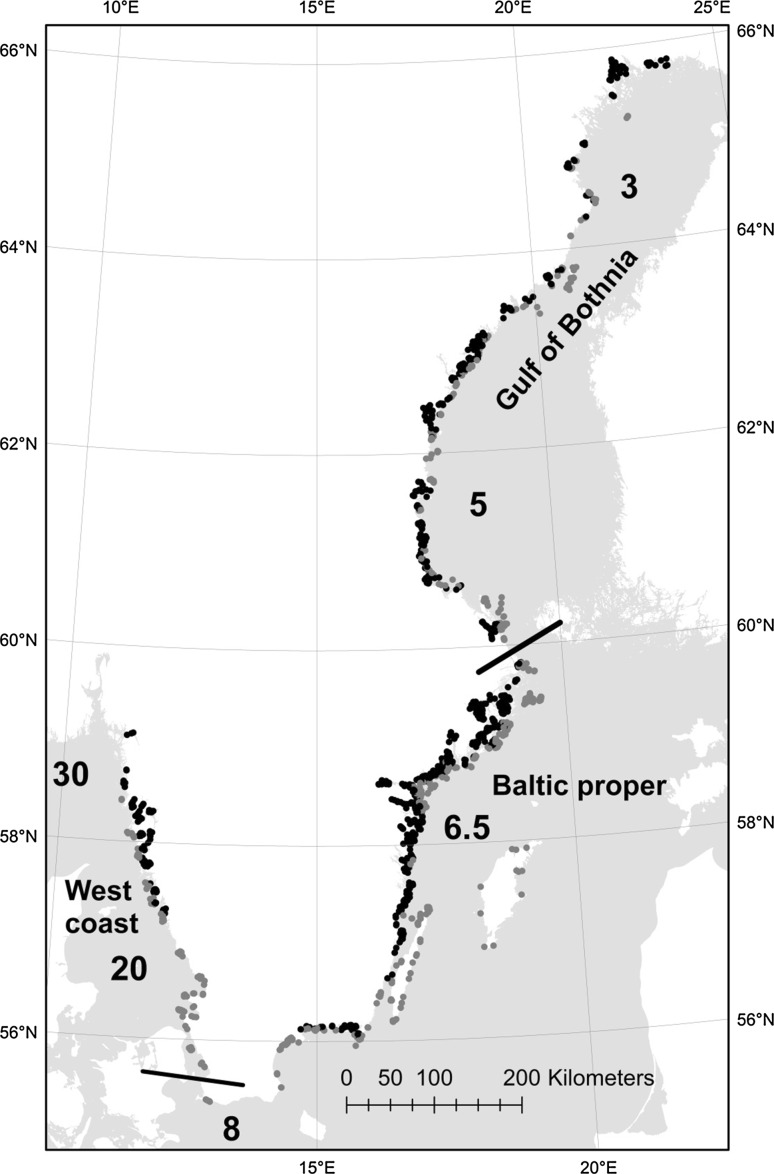
Map of study area indicating the three regions “West coast”, “Baltic Proper” and “Gulf of Bothnia”, each subdivided into inner (*black symbols*) and outer (*grey symbols*) regions. Together these represent 248 areas with data on macroalgal cover on hard substratum and 178 areas with data on cover of vascular plants and charophytes on soft/sandy substratum. Surface salinity of the open waters of various basins is indicated by *numbers*

The study included data from the entire Swedish coastline spanning 11,500 km mainland coastline (the coastline including islands >25 m^2^ is 43,400 km) from the more saline west coast (Skagerrak and Kattegat) to the brackish Baltic Sea on the east coast (comprising the sub-areas Baltic Proper and Gulf of Bothnia, Fig. 1). We divided the Swedish coast into three main regions along the salinity gradient: “West coast”, “Baltic Proper” and “Gulf of Bothnia”, each of which was divided into “inner coastal waters” and “outer coastal waters” to yield a total of six regions. The division into inner and outer coastal waters was based on the national Swedish typology (Swedish national regulation NFS 2006:1) used in the implementation of the Water Framework Directive (WFD) (2000/60/EC) in Sweden. In the statistical analyses, we also included the water bodies used in the Swedish WFD implementation as a factor in the models. The water bodies (hereafter “areas”) are defined based on coastal morphometry and water exchange and represent morphometrically delineated areas.

### Vegetation data

Vegetation data include cumulated cover of macroalgae on hard substratum and cumulated cover of vascular plants and charophytes on soft and sandy substratum. Cumulated cover refers to the summed cover of all present species and is, for simplicity, hereafter referred to as “cover”. Data originates from a range of Swedish national and regional benthic vegetation monitoring programmes which for the first time are compiled and analysed jointly. Most of the data are available in the Swedish national database for marine environmental data (available in SHARK, Svenskt HavsARKiv, www.smhi.se). In order to obtain as large and uniform a data set as possible, we selected data collected by the most commonly applied method, i.e. diving along transect lines with combined recordings of vegetation cover and substratum composition (the national standard method for the east coast of Sweden; Kautsky 1993). In this method, the transects are placed perpendicular to the shoreline, from the shallow inshore waters to the deepest occurrence of vegetation. The cover of all macroscopic algal and plant species, as well as substrate (cover of rock, boulders, stones, pebbles, sand and soft substrate), is recorded by divers in transect segments using a 7-grade cover scale (1, 5, 10, 25, 50, 75 and 100%). Recordings are conducted from deeper towards shallower depths with a new segment starting when species or substratum composition changes. Segments thus describe different vegetation zones with homogenous species and substrate composition, which differ in length and span different depth intervals. For the analyses, we assigned each segment the mean depth of the segment, calculated as the mean of the depths recorded in the deep and shallow end of the segment. This effectively meant transforming the segment data into point data describing the vegetation at the centre of the recorded segment.

### Environmental data

Data on salinity, Secchi depth and chlorophyll a and nutrient concentrations (total nitrogen and total phosphorus) originate from Swedish national and regional monitoring programmes and were obtained from the database SHARK via SMHI (www.smhi.se) on 15 December 2013. Similar water quality data covering coastal regions in the northern Baltic Proper were obtained from Svealands Kustvattenvårdsförbund (www.skvvf.se) on 22 November 2013. We used data from surface waters (average for 0–10 m depth) during the growth season (May–September) and for the same years as vegetation was sampled to characterize the environmental conditions of the investigated areas.

Physico-chemical data were linked to the positions of the vegetation data using an iterative routine that selected all stations with physico-chemical measurements within increasing distances from the vegetation transect site coordinate from the same year. For inner coastal waters, the routine searched 1, 2 or 5 km away from the site and for outer coastal waters 1, 5, 20 or 55 km away, primarily within the given water body. After at least two physico-chemical stations were found, the routine stopped and the median of the physico-chemical values was associated with the transect. Transects that did not have at least two physico-chemical stations within the chosen distances were excluded from the analyses. This method for linking physico-chemical and vegetation data was the best available option, since the monitoring programmes are not designed to always measure these variables in the same places. However, the uncertainty associated with this linkage is likely small compared to the large-scale variation among areas along the Swedish coastline.

Wave exposure was calculated with a 25 × 25 m resolution by a simplified wave model (SWM) (Isæus 2004). The model integrates the fetch in angular sectors around focal points by grid-based searches for nearby land and local mean wind speed from 16 directions. The mean wind speed was calculated for a 10-year period (1990–2000), using data from 13 wind stations distributed along the coast. All vegetation sites were assigned the SWM value from the grid cell closest to the transect site coordinate.

### Data selection

As a next step, we set up criteria to exclude segments where the vegetation cover could be expected to be regulated mainly by either availability of suitable substrate or by physical exposure. Substrate composition is a strong determinant of vegetation composition and cover, and in order to reduce the effect of differences in substrate, we only included transect segments with either homogenous hard (for macroalgae) or soft substrates (for soft-substrate vegetation). For the macroalgal cover data, we excluded segments with less than 75% cover of hard substrate (solid rock, boulders or non-mobile stones), and for the soft-substrate vegetation data, we excluded segments with less than 75% cover of soft/sandy substratum (≥75% cover of sand or smaller fractions). We further excluded observations from shallow depths where physical exposure creates large variability and can be more important than light availability in regulating cover. In order to do this, the vegetation data were divided into seven exposure classes ranging from ultra-sheltered to very exposed and plots of log-transformed cover versus depth, supported by generalized additive models, for these classes were used to determine the depth below which cover started to decline towards deeper water. This was used as cut-off depth for all transect in the exposure class (Table 1), and only observations deeper than this cut-off depth were used in the study.

**Table 1:** Exposure classes and associated depth cut-off values

Description	SWM range	Depth cut-off (m)
Ultra-sheltered	0 < SWM < 1200	0.5
Extremely sheltered	1200 < SWM < 4000	0.5
Very sheltered	4000 < SWM < 10,000	1.0
Sheltered	10,000 < SWM < 100,000	3.0
Moderately exposed	100,000 < SWM < 500,000	5.0
Exposed	500,000 < SWM < 1,000,000	7.0
Very exposed	SWM > 1,000,000	7.0

Exposure is calculated according to a simplified wave model (SWM) by Isæus (2004)

Classification and description represent preliminary Eunis classes from Wennberg and Lindblad (2006)

**Fig. 2:**
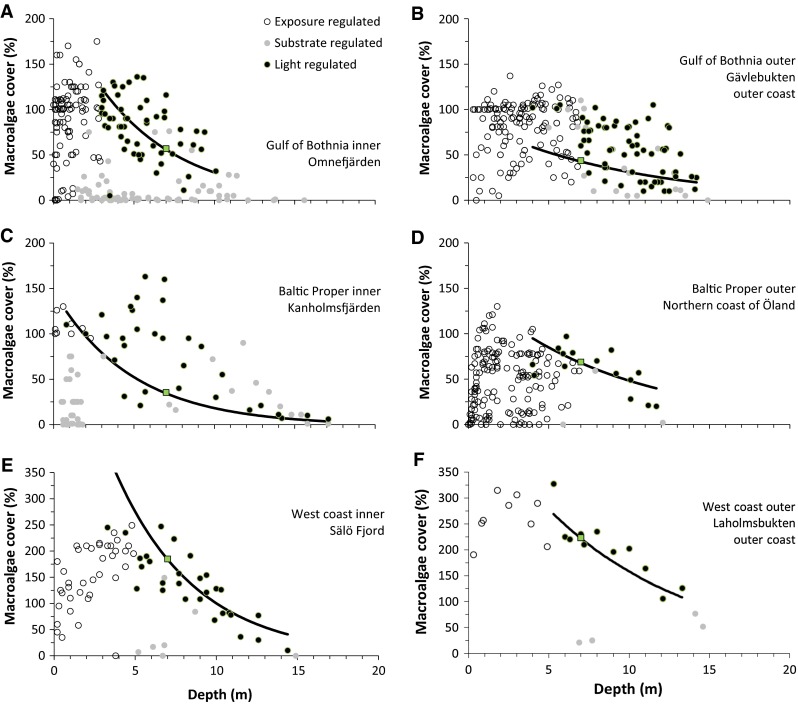
Macroalgal cover versus depth for six different areas, each representing inner and outer coastal waters for each of the three regions. Macroalgal cover is partitioned into exposure-regulated (above the cut-off depth, Table 1), substrate-regulated (<75% hard substrate) and light-regulated observations, where only the light-regulated observations were included in the analyses. Cover observations represent multiple years and multiple sites within the area, assessed by different divers. The estimated depth relationship from Eq. (1), representing an average over all years, sites and divers, is shown with a *solid line* and the estimated macroalgal cover at the standard depth of 7 m is shown with a *square*

**Fig. 3:**
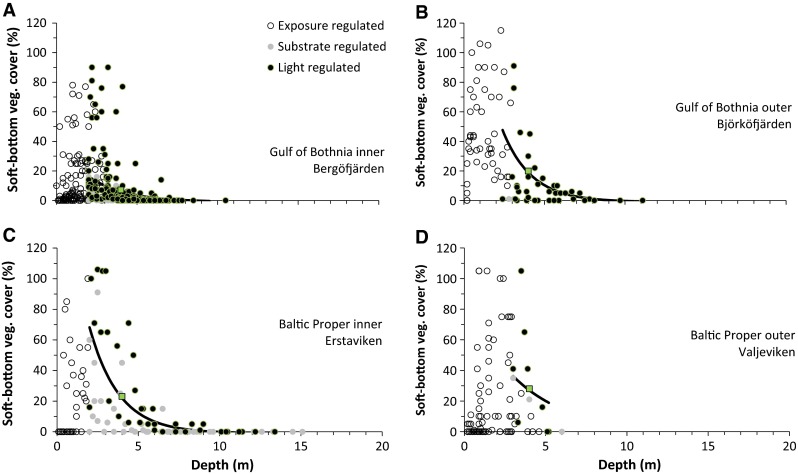
Cover of soft-substrate vegetation versus depth for four different areas, each representing inner and outer coastal waters in the Gulf of Bothnia and Baltic Proper. Soft-substrate vegetation cover is partitioned into exposure-regulated (above the cut-off depth, Table 1), substrate-regulated (<75% soft substrate) and light-regulated observations, where only the light-regulated observations were included in the analyses. Cover observations represent multiple years and multiple sites within the area, assessed by different divers. The estimated depth relationship from Eq. (1), representing an average over all years, sites and divers, is shown with a *solid line*, and the estimated cover of soft-substrate vegetation at the standard depth of 4 m is shown with a *square*

The data selection resulted in a data set of transect segments where we expect that light should be an important regulating factor for cumulative cover (e.g. Krause-Jensen et al. 2007b). We illustrate the selection process with examples from each of the six macroalgal study regions (Fig. 2) and four study regions for soft-substrate vegetation (Fig. 3). The two west coast regions were not included in the analyses of soft-substrate vegetation since very little soft-substrate vegetation data were available for these regions.

The resulting data sets on vegetation cover and environmental variables covered the entire Swedish coast and included both inner and outer coastal regions (Fig. 1). The range of environmental variables is shown in Table 2. The data set on macroalgal cover included a total of 11,932 observations in transect segments distributed across a total of 1160 sites (transects) in 248 areas (i.e. water bodies) in the 6 study regions. The considerably smaller data set on soft-substrate vegetation included a total of 3381 observations representing a total of 536 sites and 178 areas in 4 study regions. Both data sets include data from June to December from the time period 2000–2013. This period was characterized by relatively small changes in the environmental variables, with the exception of increasing phosphorus levels in some coastal areas (Moksnes et al. 2015). We therefore focus our analyses on spatial gradients in the environmental variables, comparing vegetation cover between areas with different environmental conditions.

**Table 2:** Range of physical–chemical conditions represented by the analyses

Variable	Unit	Gulf of Bothnia		Baltic Proper		West coast	
		Inner	Outer	Inner	Outer	Inner	Outer
Latitude	°N	60.1–65.9	60.1–65.3	56.1–59.8	55.4–59.8	57.3–59.1	55.7–58.4
Exposure		66–939,810	5–742,049	1–984,873	1–641,158	136–311,641	6299–984,873
Salinity		0.5–5.8	0.6–5.3	1.8–7.8	5.9–8.3	18.4–25.9	9.2–30.5
Secchi depth	m	1.0–8.5	3.0–8.0	1.3–10.5	5.0–12.0	3.0–8.3	4.5–9.0
Total nitrogen	µmol L^−1^	11.3–76.3	11.2–22.7	15.0–52.7	17.5–25.4	14.9–20.9	12.0–19.4
Total phosphorus	µmol L^−1^	0.16–2.40	0.14–0.51	0.38–2.25	0.33–1.13	0.33–0.88	0.36–0.78
Chlorophyll a	µg L^−1^	1.3–41.6	1.4–11.1	0.8–15.0	0.5–9.6	1.3–4.4	0.7–3.2

Values represent growth season (May–September) surface (0–10 m depth) values for all variables except latitude and exposure. Exposure is calculated according to a simplified wave model (SWM) by Isæus (2004)

### Statistical analyses

The statistical analyses were done in two steps. First, we first ran linear mixed models (LMM) of vegetation cover to account for sampling-specific variation (year, month, site, depth and diver) and produce comparable mean estimates of vegetation cover for the 248 areas with macroalgae data and 178 areas with data on vascular plants and charophytes. The models were fitted using residual restricted maximum likelihood estimation with hierarchical model comparison by *F*-test for fixed effects and asymptotic Wald *Z*-test for variances of random effects. We ran separate models for each of the six regions defined in the study (outer and inner coast of the west coast, Baltic Proper and Gulf of Bothnia; Fig. 1) since we expected different seasonal patterns and trends over time between northern and southern Sweden and between inner and outer coastal areas. Mean log-transformed cover (*µ*_*ijk*_) in each region was described as:

1 $$\mu_{ijk} = {\text{area}}_{i} + {\text{year}}_{j} + {\text{month}}_{k} + \beta \cdot {\text{depth}}$$

where area_*i*_ describes the differences between areas, year_*j*_ describes differences between years, month_*k*_ describes differences between months of sampling and $\beta \cdot {\text{depth}}$ describes the decline in cover with depth (*β* is the slope for the log-transformed cover observations). In addition, site within area and diver (the person performing the vegetation survey) were included as random factors. We analysed log-transformed cover since the residuals from Eq. (1) of both macroalgae and soft-substrate vegetation were approximately normally distributed after log transformation. Hence, the linear depth relationship for the log-transformed observations reflects an exponential decrease in cover with depth as expected from the attenuation of light with depth (Duarte 1991). Variation between months (June–December) was not significant for any combination of region and macroalgae/soft-substrate vegetation, and this factor was therefore excluded from the models.

For each region, marginal means for areas and years were computed from the parameter estimates of Eq. (1) by averaging over the parameters of qualitative factors in the equation and predicting for depths of 4 m for cover of soft-substrate vegetation and 7 m for cover of macroalgae. These depths of standardization, which were within the observation depth ranges in most areas, were chosen to produce comparable marginal means for areas and years. For example, the area-specific marginal mean for macroalgae cover represents an average across all years with data from that region at a depth of 7 m. There were also differences between regions in which years cover had been monitored, but the inter-annual variations were generally small, compared to depth variations, and these differences would only marginally influence the comparison of area-specific means between regions.

The area-specific mean cover estimates from these first models were used in the second step of the analyses, where we investigated the potential regulation of vegetation cover by environmental factors. In this step, the area-specific mean cover of macroalgae and soft-substrate vegetation was modelled as a function of mean salinity, TN or TP, chlorophyll and Secchi depth from monitoring data and modelled wave exposure (SWM). Area-specific means of salinity, TN, TP, chlorophyll and Secchi depth were estimated by first averaging over summer (May–September) observations from the area for each year where vegetation was also monitored and then averaging over all years with vegetation data. In addition, the mean latitude of the vegetation transects in the area was included as photoperiod/irradiance was also hypothesized to influence vegetation cover. In this analysis, we looked for general patterns across the entire Swedish coastline and consequently analysed data from all six regions in one single model. We employed a spline function within the generalized additive model (GAM) framework for testing for higher-order relationship in addition to a linear model, since the exact nature of the putative relationships was not known. Smoothing was determined by generalized cross-validation, but to reduce the curvature of the relationships a maximum of 3 degrees of freedom was imposed for each of the explanatory variables in the GAM model. Environmental factors were included only if they explained a significant proportion of the variation in addition to the other explanatory factors (i.e. by comparing the model with and without the given factor using likelihood ratio test). This model selection approach reduced the potential effect of inter-correlation between the environmental factors, and the marginal relationship for the different explanatory variables (i.e. adjusting for the other factors) was plotted to assess its nature and potential sensitivity to potential outliers. If the higher-order relationship was not significant, the relationship with the explanatory variable was reduced to a linear relationship and tested again. Through this backward elimination procedure, non-significant nonparametric smoothers and linear relationships were iteratively excluded until all factors included in the model were significant. All statistical analyses were carried out in SAS 9.3 using PROC MIXED and PROC GAM.

## Results

### Sources of variation in estimates of vegetation cover

For macroalgae on hard substrate, there were large differences in the number of observations and, hence, in monitoring effort between the six regions, ranging from 626 observations in west coast inner to 3888 observations in Baltic Proper inner (Table 3). Macroalgal cover decreased significantly with depth in all regions, with a faster decrease (steeper regression slope) in the inner compared to the outer areas. There was also significant spatial variation between sites and areas in all regions and also between divers in two of the six regions (Table 3). Spatial variation between transects and residual variation around the depth relation were considerably larger than diver-specific variation (Table 4). In fact, cover estimates for individual segments could vary by factors 1.5–8 along the depth gradient (Table 4), illustrating the large variability in these data. Variation between sites within an area varied from 41% for macroalgae in west coast outer to 129% for macroalgae in Baltic Proper outer. Variation in macroalgae cover between sites was generally lower along the saline west coast of Sweden, compared to the brackish Baltic Proper and Gulf of Bothnia. Variation between divers was also considerable, suggesting that cover estimates vary by approximately 50% when different divers monitor the same site (Table 4). Inter-annual variation in macroalgae cover was significant in five of the six study regions, and two of these displayed a significant trend over time with cover levels increasing in Baltic Proper inner (linear regression; *P* = 0.0008) and west coast inner (linear regression; *P* = 0.0182) (Online Resource 1). It should also be noted that macroalgal cover was substantially higher in the outer as compared to the inner regions and that estimates from regions with few data were associated with large uncertainty.

**Table 3:** Test of fixed and random factors for the linear mixed models of cover of macroalgae and soft-substrate vegetation (log-transformed values)

Region	Number of observations (years)	Fixed factors			Random factors	
		Area	Year	Depth	Site	Diver
*Macroalgae cover*						
Gulf of Bothnia inner	1064 (13)	**<0.0001**	**0.0288**	**<0.0001**	**<0.0001**	0.1091
Gulf of Bothnia outer	1215 (13)	**0.0020**	0.5835	**<0.0001**	**<0.0001**	0.0807
Baltic Proper inner	3888 (14)	**<0.0001**	**0.0003**	**<0.0001**	**<0.0001**	**0.0121**
Baltic Proper outer	3859 (14)	**<0.0001**	**<0.0001**	**<0.0001**	**<0.0001**	**0.0150**
West coast inner	626 (6)	**<0.0001**	**<0.0001**	**<0.0001**	**<0.0001**	0.2663
West coast outer	1349 (6)	**0.0089**	**0.0001**	**<0.0001**	**<0.0001**	0.1467
*Soft*-*substrate vegetation cover*						
Gulf of Bothnia inner	943 (12)	**<0.0001**	0.1885	**<0.0001**	**<0.0001**	0.0991
Gulf of Bothnia outer	176 (11)	0.4728	0.1984	**<0.0001**	**0.0045**	0.1771
Baltic Proper inner	1854 (14)	**<0.0001**	0.4694	**<0.0001**	**<0.0001**	**0.0225**
Baltic Proper outer	408 (12)	0.7663	0.4832	**<0.0001**	**0.0076**	**0.0442**

Significance of fixed effects was tested with *F*-test and of random effects with asymptotic Wald *Z*-test

Significant factors (*P* < 0.05) are highlighted in bold

**Table 4:** Regression slope (depth) and variance estimates for the random factors and their relative contribution to the uncertainty associated with individual segment observations of cover of soft-substrate vegetation

Region	Slope for depth	Variance estimates			Relative uncertainty		
		Site	Diver	Residual	Site (%)	Diver (%)	Residual (%)
*Macroalgae cover*							
Gulf of Bothnia inner	−0.195	0.5973	0.0761	2.9342	117	32	455
Gulf of Bothnia outer	−0.104	0.6882	0.1789	4.6862	129	53	771
Baltic Proper inner	–0.204	0.4343	0.1009	2.3636	93	37	365
Baltic Proper outer	−0.111	0.4441	0.0478	2.7812	95	24	430
West coast inner	−0.201	0.2500	0.0435	0.8276	65	23	148
West coast outer	−0.113	0.1174	0.1431	0.8003	41	46	145
*Soft*-*substrate vegetation cover*							
Gulf of Bothnia inner	−0.438	0.3541	0.1138	5.4000	81	40	921
Gulf of Bothnia outer	−0.559	0.4858	0.3161	5.3020	101	75	900
Baltic Proper inner	−0.551	0.4227	0.1119	4.9460	92	40	824
Baltic Proper outer	−0.286	0.3270	0.6859	6.5139	77	129	1184

The relative uncertainty was calculated as $\exp \left( {\sqrt V } \right) - 1$, based on lognormal distribution theory

Cover of soft-substrate vegetation was analysed in the two Gulf of Bothnia regions as well as in the two Baltic Proper regions but not in the west coast regions where too few observations were available. Overall, there were fewer observations on soft-substrate vegetation and therefore less data available for estimating the different sources of variation as compared to the macroalgae. This resulted in fewer significant estimates of the sources of variations (Tables 3, 4). The most important sources of variation were water depth and random variation between sites (transects), which were consistently significant (Table 3). Cover of soft-substrate vegetation decreased significantly with depth in all regions, with a faster decrease (steeper regression slope) compared to the macroalgal vegetation. Differences between areas were larger for inner than for outer coastal regions and significant for the inner regions only. Variation between divers was significant for the Baltic Proper but not for the Gulf of Bothnia. The random variation between transects was similar to that for macroalgae, but the residual variation, expressing the patchiness in soft-substrate vegetation cover, was much higher. None of the regions showed any significant inter-annual variation in the cover of soft-substrate vegetation (Table 3, Online Resource 1).

### Relationships between vegetation cover and environmental variables

**Fig. 4:**
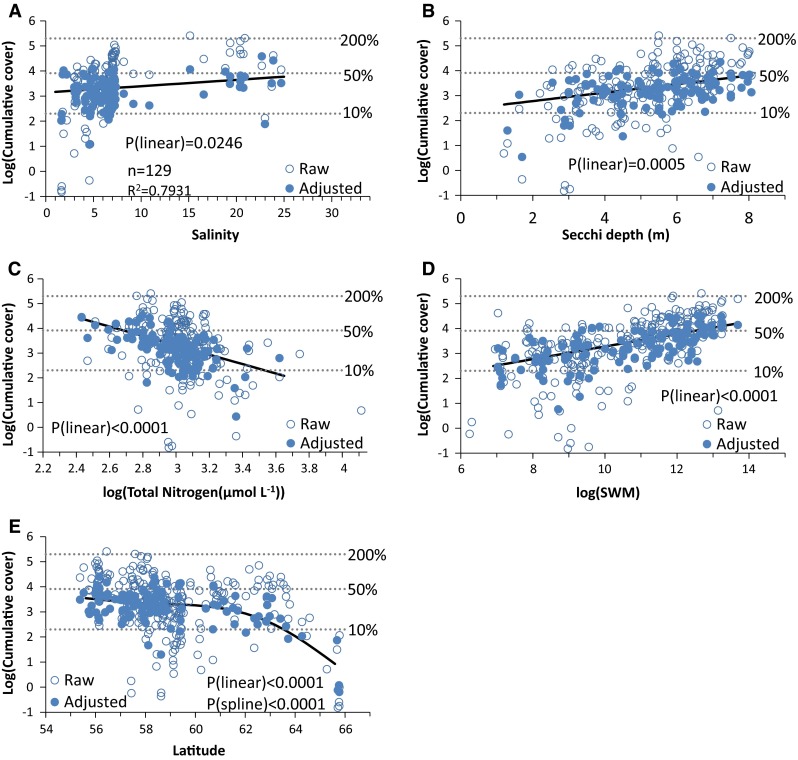
GAM relationships between area-specific means of macroalgae cover (log-transformed) and environmental variables obtained from monitoring data (only 129 areas had data on all environmental variables). *Open symbols* show the area-specific means (raw), and *filled symbols* show the means adjusted for variations explained by the other four factors in the GAM model. Expected mean cover was adjusted to average salinity of 6.5, Secchi depth of 5.1 m, log (SWM) of 10.1, log (TN) of 3 and latitude of 59°N. Adjusted means for cover could only be calculated for areas where data on all environmental variables were available. Statistics for the GAM are inserted in the salinity plot. For readability, three back-transformed cumulative cover levels (10, 50 and 200%) are shown with *dotted lines*

The final model of average macroalgal cover at 7 m depth included five environmental variables (salinity, Secchi depth, TN concentration, exposure and latitude) which together explained 79% of the variation in cover between areas (Fig. 4). The log-transformed cover estimates increased linearly with salinity (slope = 0.0252; *P* = 0.0241), and the expected cover at 7 m increased from 23 to 49% along the studied salinity gradient. Macroalgae cover also increased with increasing Secchi depth (slope = 0.172; *P* = 0.0005) from 14% at a Secchi depth of 1.1 m to 46% at Secchi depth of 8 m. In addition, cover increased with reduced TN concentration (slope = −1.89 for log (TN); *P* < 0.0001) with a predicted decline of cover from 103% at a TN concentration of 10 µmol L^−1^ to 4% at a concentration of 60 µmol L^−1^. Physical exposure had a significantly positive effect on macroalgae cover (slope = 0.252 for log (SWM); *P* < 0.0001); the physical exposure caused an increase in cover from 10% at the most sheltered to 67% at the most exposed sites. Macroalgae cover had a higher-order relationship to latitude (*P* < 0.0001 for linear component as well as for smoother) with only a small decrease in predicted cover as a function of latitude for latitudes below 62°N and a steeper decrease at higher latitudes. The rapid decline in the northernmost part of the gradient was to a large extent driven by very low cover in most areas in the Bothnian Bay (north of 64°N).

**Fig. 5:**
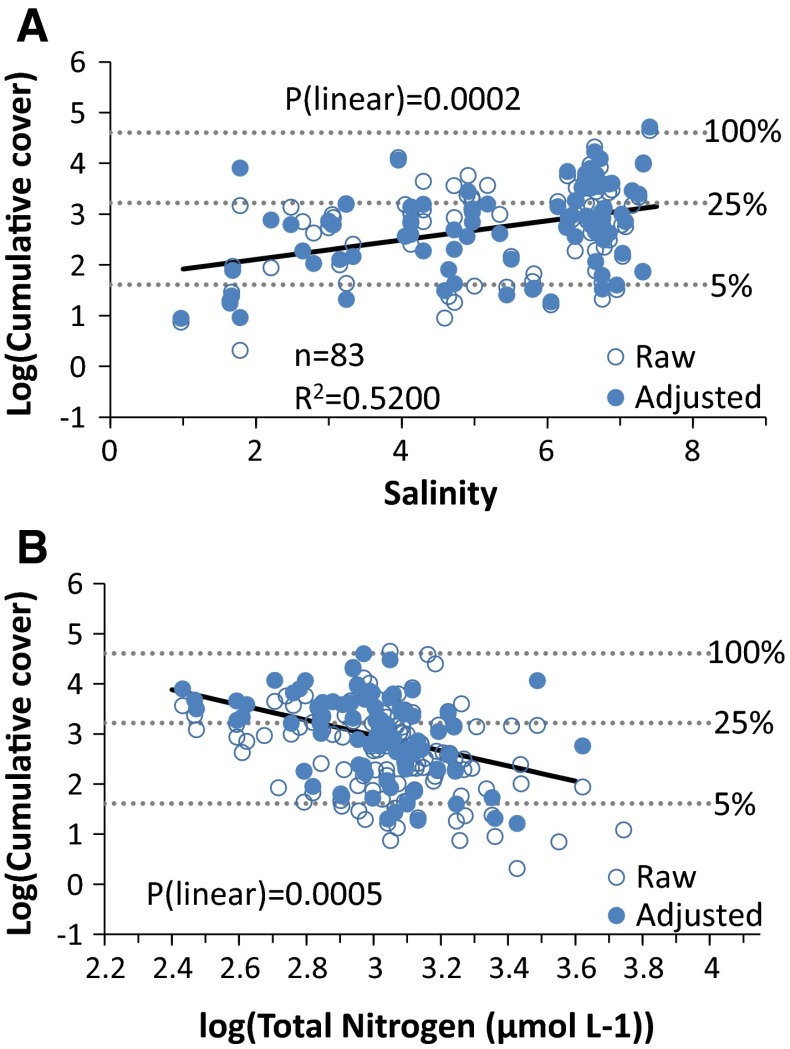
GAM relationships between area-specific means of cover of soft-substrate vegetation (log-transformed) and environmental variables obtained from monitoring data (only 83 areas had data on all environmental variables). *Open symbols* show the area-specific means (raw), and *filled symbols* show the means adjusted for variations explained by the other four factors in the GAM model. Expected mean cover was adjusted to average salinity of 6.5 and log (TN) of 3. Adjusted means for cover could only be calculated for areas where data on all environmental variables were available. Statistics for the GAM are inserted in the salinity plot. For readability, three back-transformed cumulative cover levels (5, 25 and 100%) are shown with *dotted lines*

The final model of average cover of soft-substrate vegetation at 4 m depth included TN concentration and salinity (Fig. 5). Overall, the model explained 52% of the variation in cover among areas, which gives a considerably lower predictability compared to macroalgae cover. The log-transformed cover decreased linearly with increasing TN concentration (slope = −1.47 for log (TN); *P* = 0.0005), yielding a difference in expected cover at 4 m depth from 57% at 10 µmol L^−1^ to 4% at 60 µmol L^−1^, when accounting for variations due to salinity. Salinity, on the other hand, had as significantly positive effect (slope = 0.192; *P* = 0.0002) on cover yielding an increase in expected cover from 6% at the lowest salinity to 38% at a salinity around 10, when accounting for the effect of TN (Fig. 5).

## Discussion

### Response of vegetation cover to gradients of eutrophication

Vegetation cover of hard and soft substrate at a defined depth (7 and 4 m depth, respectively) increased significantly towards areas with low nutrient concentration and high water clarity, thereby reflecting gradients in eutrophication across the Swedish coastline. These findings support and expand earlier studies from parts of the Baltic Sea (Krause-Jensen et al. 2007a, b, 2009; Hansen and Snickars 2014) by highlighting that similar relationships between vegetation cover and eutrophication-related physico-chemical variables operate across the broad environmental gradients in the Baltic Sea, Kattegat and Skagerrak. The relationships are also in accordance with findings from other shallow aquatic ecosystems where increased nutrient richness generally stimulates the proliferation of epiphytes and drifting opportunistic algae and increase water column light attenuation, thereby hampering the benthic vegetation (Sand-Jensen and Borum 1991; Duarte 1995; Cloern 2001; Krause-Jensen et al. 2007a, b). Eutrophication effects on light-attenuating components of the water column can be complex and may involve stimulation of phytoplankton biomass (e.g. Nielsen et al. 2002) as well as increased resuspension of particles, e.g. after loss of vegetation cover (Carr et al. 2010; Carstensen et al. 2013) and increased levels of dissolved organic matter related to increased production (Pedersen et al. 2014). Factors unrelated to eutrophication also affect light attenuation and may interact with eutrophication-related effects. Particularly, the most brackish waters furthest north in the Baltic Sea are influenced by terrestrial run-off with high concentrations of dissolved organic matter, which reduces light levels even though bioavailable nutrient levels are low (Fleming-Lehtinen and Laamanen 2012; Tolvanen et al. 2013).

The relationships between vegetation cover and eutrophication-related variables appeared after accounting for variation in vegetation cover due to other factors such as salinity, exposure and/or latitude as well as for variation caused by differences in sampling depth and substrate characteristics, sampling season and differences between divers. This highlights that knowledge on natural sources of variability affecting vegetation cover increases the chance to detect a response to pressure, in this case eutrophication. Including the natural gradients allowed establishment of a general relationship between vegetation cover and eutrophication variables throughout all study areas.

The relationship between increased vegetation cover and reduced nutrient concentrations was documented both for macroalgae on hard substrate and vascular plants and charophytes on soft substrate. However, the empirical model describing vegetation cover as function of environmental variables was stronger and more robust for macroalgal vegetation than for soft-substrate vegetation. The models explained 79% of the variation in macroalgal cover by a combination of variables related to eutrophication (TN concentration and Secchi depth), salinity, exposure and latitude. By contrast, only 52% of the variation in soft-substrate vegetation could be explained by environmental variables, and in this case, only TN concentration and salinity contributed significantly to explaining the variation. The weaker empirical relationship for soft-substrate vegetation may in part be due to a smaller data set which decreased the potential for partitioning the different sources of variability and increased the uncertainty of the area-specific mean cover estimates. In fact, the large small-scale spatial variability that we documented for the soft-substrate vegetation suggests that a much larger sampling effort is required for soft-compared to hard-substrate vegetation in order to achieve a comparable certainty in the estimated mean cover. However, we cannot exclude that the cover of soft-substrate vegetation is also affected by factors not included in the model. For instance, sediment characteristics such as grain size distribution and organic content can have a large impact on growth and species composition of aquatic vegetation (Koch 2001).

### Vegetation response to natural gradients

The cover of macroalgae showed a significantly positive coupling to the strong gradient in salinity along the Swedish coastline. This may be related to the increase in the number of species in general and of large canopy-forming species in particular with increasing salinity from the Swedish northeast coast in the inner parts of the Baltic Sea to the Kattegat (Nielsen et al. 1995). The presence of kelps and other large engineering species in the marine waters stimulates the formation of multi-layered communities with canopies and understorey macroalgal vegetation, increasing cumulative cover as well as habitat diversity and consequently species diversity (Gutiérrez et al. 2011). Also the cover of soft-substrate vegetation was positively related to salinity. However, in this analysis salinity was strongly correlated with latitude, since the analysis did not include data from the west coast of Sweden, and thus represents a gradient in both salinity and climatic conditions. The increase in cover with salinity cannot be explained by increasing diversity, since the diversity of vascular plants and charophytes is highest in areas with low salinity (Boström et al. 2014). It is possible that the increase in soft-substrate vegetation cover is related to the appearance of eelgrass at salinities above ca. 5, since this species extends deeper and into more exposed habitats than most other vascular plants. However, the low cover in the low-saline Bothnian Bay may also be explained by, for example, winter darkness and extended ice cover reducing the period with sufficient light to support growth. Further studies of the response to salinity within a restricted latitudinal range could help untangle the separate effects of salinity and climate on cover of soft-substrate vegetation.

Macroalgal cover decreased with latitude, with the most prominent decline north of 62°N. This decline may, as discussed above for the soft-substrate vegetation, reflect longer periods of darkness and ice cover in the north. The extent of ice cover differs between years, but the Bothnian Bay is covered every year and has the longest duration of ice cover (on average >150 days; Al-Hamdani and Reker 2007). Increased algal cover and broader vegetation belts have also been reported in the Arctic as a response to longer ice-free periods (Krause-Jensen et al. 2012; Kortsch et al. 2012). Hence, in addition to small-scale effects of light along depth gradients and medium-scale effects between areas differing in water column light attenuation, the vegetation may also respond to large-scale light gradients related to differences in the ice-free period and photoperiod.

Macroalgal cover was also positively related to wind-generated exposure after excluding data from the shallowest parts of the transects, where negative effects of wave exposure are observed. The positive effect of exposure could be that waves increase water and macroalgae motions at deeper depths, enhancing the supply of micro- and macro-nutrients (Hurd 2000). Water movement also reduces sedimentation of particles on the sea floor and macrophyte surfaces, which can in turn increase establishment and growth of macroalgae (e.g. Isæus et al. 2004; Eriksson and Johansson 2005). Similar positive effects of exposure could be expected for soft-substrate vegetation up to moderate exposure levels below the tolerance threshold of eelgrass and other soft-substrate vegetation (Koch 2001), but could not be detected in this study.

### Development of an indicator for vegetation cover

The clear effect of a number of natural environmental gradients on vegetation cover shows that these gradients have to be accounted for when developing cover as an indicator for ecological quality of coastal areas. One possible approach is to use the models developed in this study to extract empirical relationships between vegetation cover and environmental variables, which can be used to compensate for variation in variables that are not connected to anthropogenic impact. The suggested method for monitoring vegetation cover is vegetation surveys at a number of sites in the area in question, with identification of cover along depth gradients focusing on water depths deeper that the cut-off depth (see Table 2) and prioritizing information on seafloors representing >75% hard substrate in the case of macroalgal surveys and >75% soft substrate in the case of soft-substrate vegetation. Links to environmental variables for the relevant area and year should also be ensured.

Temporal responses of ecosystem status to changes in pressures can exhibit considerable complexity, for example, related to resilience of ecosystem states which may result in some divergences between predictions and reality and may introduce lags in response (Duarte et al. 2007; Carstensen et al. 2013; Riemann et al. 2016). Vegetation responses to environmental controls are, for example, likely to follow different pathways during periods of decline and recovery due to feed-back effects of the vegetation (e.g. van der Heide et al. 2011; Maxwell et al. 2016). A dense vegetation can to some extent buffer and delay potential negative eutrophication effects, for instance by improving water clarity through interception of nutrients and trapping of sediments(Carr et al. 2010), and by providing habitat for fish that can control epiphyte cover (Baden et al. 2010; Bennett et al. 2015; Maxwell et al. 2016). By contrast, recovery of lost vegetated habitats may be delayed because of resilience of the bare state where nutrients are primarily taken up by phytoplankton, resuspension of sediments contribute to maintaining reduced water clarity, and top-down controls may be disrupted (Krause-Jensen et al. 2012; Carstensen et al. 2013; Duarte et al. 2013). It is, therefore, important to note that while the data set used in the present study represented a time span of 13 years, temporal differences in environmental variables were very small and only few trends in cover were observed over the study period. Thus, although the predictive models established in the current study include ecosystems in various stages, it is possible that they do not fully capture vegetation response to future changes in, for example, eutrophication levels. We therefore suggest that the models are updated in the future when time series with changes in eutrophication variables becomes available.

While we show that vegetation cover is a promising indicator that responds to water quality, it does not capture all important aspects of healthy coastal vegetation. In theory, the vegetation cover could remain intact also when the species composition changes, for instance when sensitive species are replaced by opportunistic macroalgae or plants. The cover indicator should therefore be complemented with an indicator of species composition. For instance, Hansen and Snickars (2014) complemented cover of soft-substrate vegetation with a community index measuring the relative abundance of species that are tolerant and sensitive to eutrophication. Similarly, the Spanish indicator for hard substrate vegetation in the Atlantic combines macroalgal cover with species richness and the presence of opportunistic species (Juanes et al. 2008).

## Conclusion

In conclusion, the results show that cover of both macroalgae and soft-sediment vegetation vary predictably over gradients in eutrophication as expressed by nutrient concentrations and water clarity, when accounting for variation due to other environmental variables as well as spatio-temporal and diver-related sampling variability in the study areas. The findings hence suggest that vegetation cover may be of general use for monitoring and management of marine vegetation, also in areas with strong natural gradients such as the Swedish coast.

## Electronic supplementary material

Below is the link to the electronic supplementary material.

Supplementary material 1 (PDF 204 kb)
