# Supplementary material for: Cover of coastal vegetation as an indicator of eutrophication along environmental gradients
Source: Mar Biol. 2016 Nov 21;163(12):257. doi: 10.1007/s00227-016-3032-6 (PMC5116445; doi:10.1007/s00227-016-3032-6)
Supplement: Supplementary file 1 — Supplementary material 1 (PDF 204 kb) [file 227_2016_3032_MOESM1_ESM.pdf]

Online Resource 1

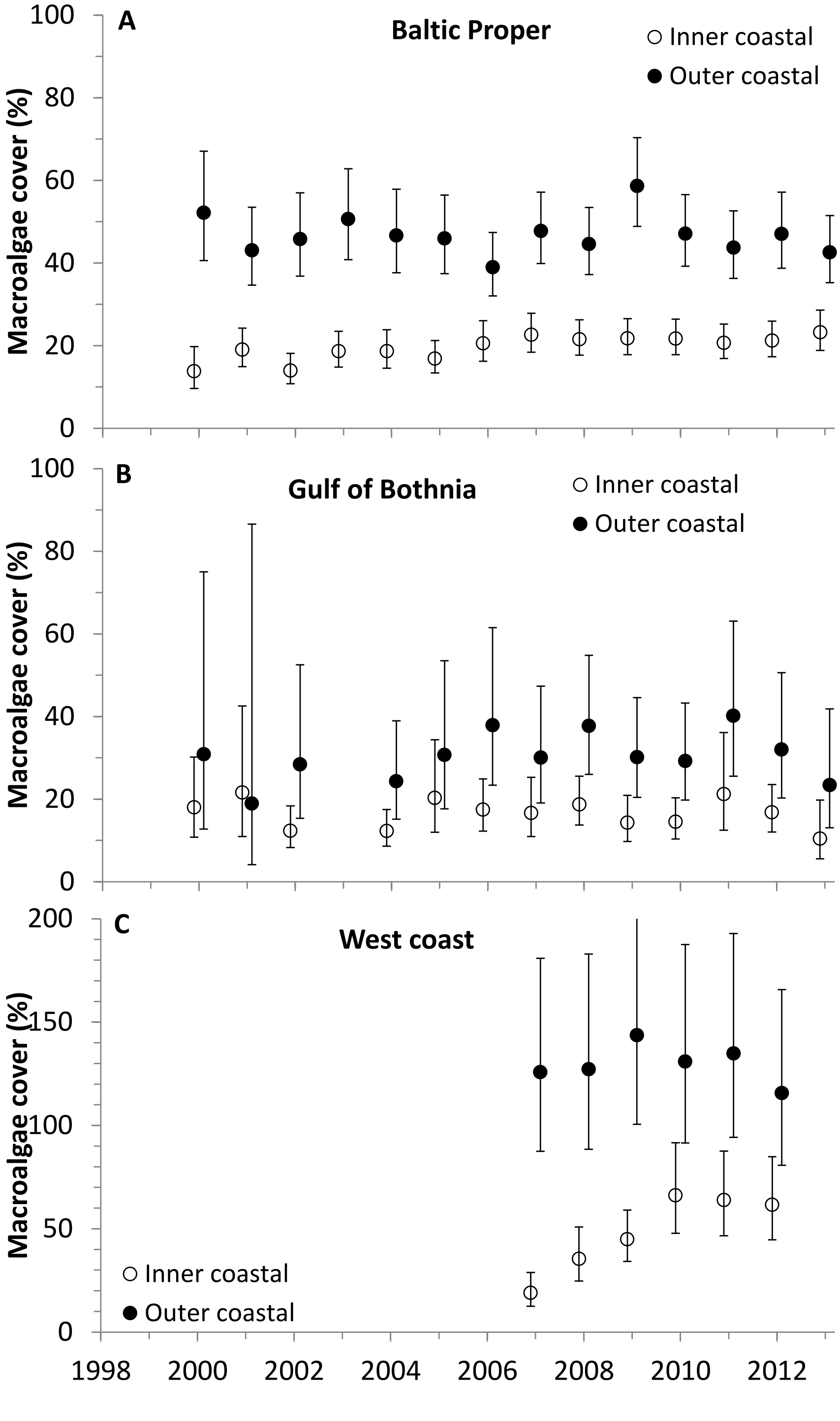

Fig. 1 Estimated inter-annual variation in macroalgal cover for inner and outer coastal areas in the three study regions. Error bars mark the 95% confidence interval for the geometric means. Note the log-scale on the y-axes

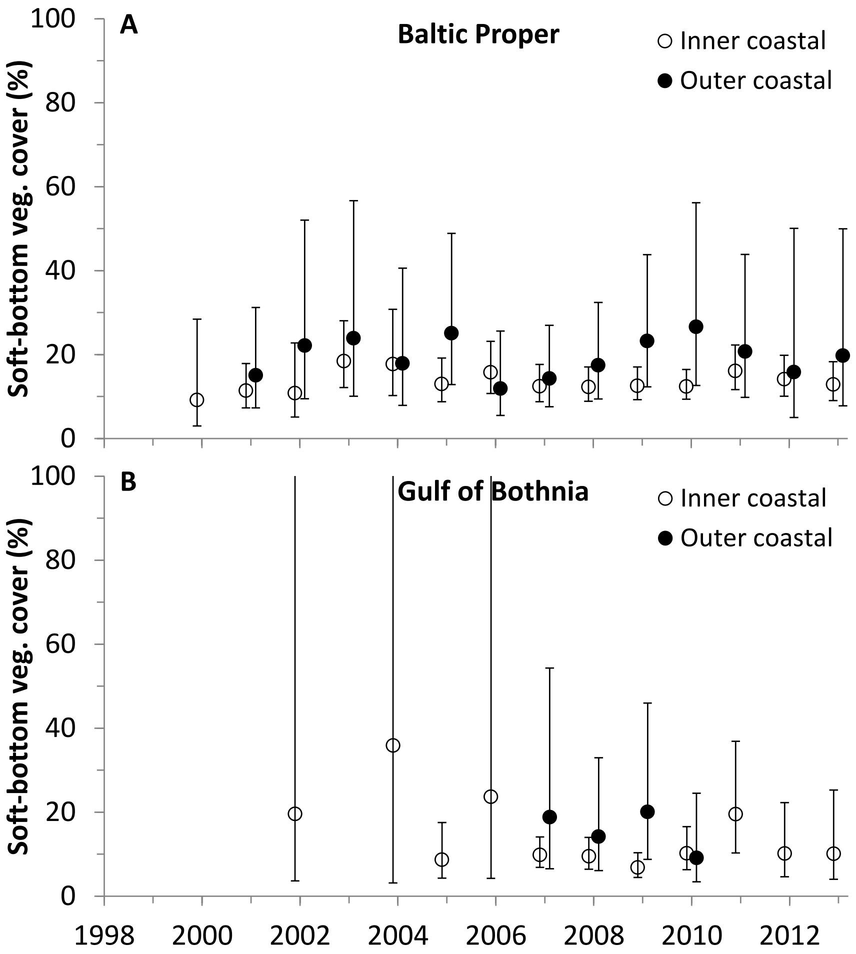

Fig. 2 Estimated inter-annual variation in cover of soft substrate vegetation for inner and outer coastal areas in the three study regions. Error bars mark the 95% confidence interval for the geometric means. Note the log-scale on the y-axes
